# First contact: an interdisciplinary guide into decoding H5N1 influenza virus interactions with glycosaminoglycans in 3D respiratory cell models

**DOI:** 10.3389/fcimb.2025.1596955

**Published:** 2025-05-15

**Authors:** Mariam Hassan, Bianca Kaifer, Tyra Christian, Xenia Tamara Quaas, Johannes Mueller, Heike Boehm

**Affiliations:** ^1^ Institute of Pharmacy and Molecular Biotechnology, Faculty of Engineering Sciences, Heidelberg University, Heidelberg, Germany; ^2^ Max Planck Institute for Medical Research (MPIMR), Cellular Biophysics, Heidelberg, Germany

**Keywords:** influenza A - subtype H5N1, glycosaminoclycans, 3D respiratory cell models, synthetic viruses, air-liquid Interface (ALI) 3D *in vitro* model, fluorescent microscopy

## Abstract

The human respiratory system is vulnerable to viral infections. The influenza virus family alone accounts for one billion reported cases annually, some of which are severe and can be fatal. Among these, Influenza A viruses (IAVs) cause the most severe symptoms and course of disease. IAV has been a major health concern, especially since the emergence of the potentially pandemic avian H5N1 strain. However, despite the knowledge that IAVs recognize terminally attached sialic acids on the host cell surface for cell entry, the involvement of other glycans during early infection remains to be elucidated. In particular, the involvement of the alveolar epithelial glycocalyx as a last line of defense is often overlooked. Studying early infection of any virus in real time remains a challenge due to the currently available model systems and imaging techniques. Therefore, we extensively compare the use of different 3D cell systems and provide an overview of currently available scaffold-based and scaffold-free air-liquid interface (ALI) models. In addition, we discuss in detail the preferred use of a recently developed 3D organ tissue equivalent (OTE) model incorporating solubilized extracellular matrix components (sECM) to study viral interaction with glycosaminoglycans (GAGs) during the early stages of IAV infection. We further discuss and recommend the use of various synthetic virus models over IAV virions to reduce complexity by focusing only on surface protein interactions while simultaneously lowering the required biosafety levels, including, but not limited to virus-like particles (VLPs) or DNA origami. Finally, we delve into potential labeling strategies for IAV or IAV-like particles by reviewing internal and external labeling strategies with quantum dots (QDs) and potential GAG labeling, combined with a recommendation to combine high spatial resolution imaging techniques with high temporal resolution tracking, such as single virus tracking.

## 1 Introduction

Oxygen uptake and carbon dioxide release are essential for the human organism and are enabled by the respiratory system. The respiratory system is targeted by many pathogens, including the influenza virus family. Within this family, the IAV virus is the causative agent of the most severe infections (Couch, 1996). In recent years, the H5N1 strain has acquired the potential to cause a new influenza pandemic (Who, 2024). Thus, appropriate models are needed to study the mechanism of infection, the subsequent pathologies, and the potential threat. Understanding the native system is critical to the development of meaningful models.

Therefore, we begin this review with a brief overview of the respiratory system, focusing on the physiological characteristics of the alveoli. We then present general information and previous findings on the pandemic risk posed by H5N1. Before discussing current and future research directions, we highlight how GAGs can be incorporated in the models, as they may be critical contributors that are often overlooked (Varki, 2017). Thereby, the involvement of the glycocalyx of the alveolar epithelium in early infection can be assessed.

We then delve deeper into the different 3D *in vitro* cell culture models and provide a baseline for choosing the right model. Additionally, we suggest novel methods for producing a synthetic virus in order to conduct experiments with lower biosafety levels. Finally, we explore visualization techniques for analyzing GAG interactions with viruses and synthetic viruses at high resolutions (Liu et al., 2023; Perez et al., 2023). We also discuss the spectrum of available tools, from fluorophores and quantum dots to advanced microscopy techniques, highlighting their applicability, limitations, and potential for customization. By combining advanced models and techniques, we encourage the study of virus-GAG interactions, ultimately contributing to a better understanding of IAV infection and the prevention of future human health risks.

## 2 The respiratory system

The human respiratory system can be divided into the air-conducting respiratory system and the respiratory zone. A specialized epithelium, coated by a mucus layer, lines the whole respiratory system. In the nasal cavity, inhaled air is heated up due to the high vascularization of the tissue and is humidified by the mucus. The mucus contains about 97% water; other components are lipids, salts, non-mucin proteins (e.g., surfactant proteins, host defense proteins), and mucins (high molecular weight glycoproteins). Mucins are cross-linking monomers that bind large amounts of fluid ensuring elastic and lubricant mucus properties (Bonser and Erle, 2017; Hill et al., 2022). The mucus is crucial for trapping potentially harmful pathogens and particles present in the inhaled air.

Cell composition and functions of the epithelium differ in the various sections of the air-conducting segments of the respiratory tract. Goblet cells produce mucus alongside the serous cells of the mucus-producing glands (Whitsett, 2018; Mescher, 2021). The beating of columnar ciliated cells traps and evicts debris and mucus (Whitsett, 2018; Mescher, 2021). Basal cells serve as progenitor cells (Whitsett, 2018; Mescher, 2021). The rare pulmonary neuroendocrine cells can sense changes in oxygen levels as well as physical and chemical stimuli. In response to these signals, they release neuropeptides and neurotransmitters to trigger immune responses and physiological effects (Linnoila, 2006; Cutz et al., 2013; Branchfield et al., 2016). Among these cells, several different cell types serve similar and additional functions.

### 2.1 The alveoli

**Figure 1:**
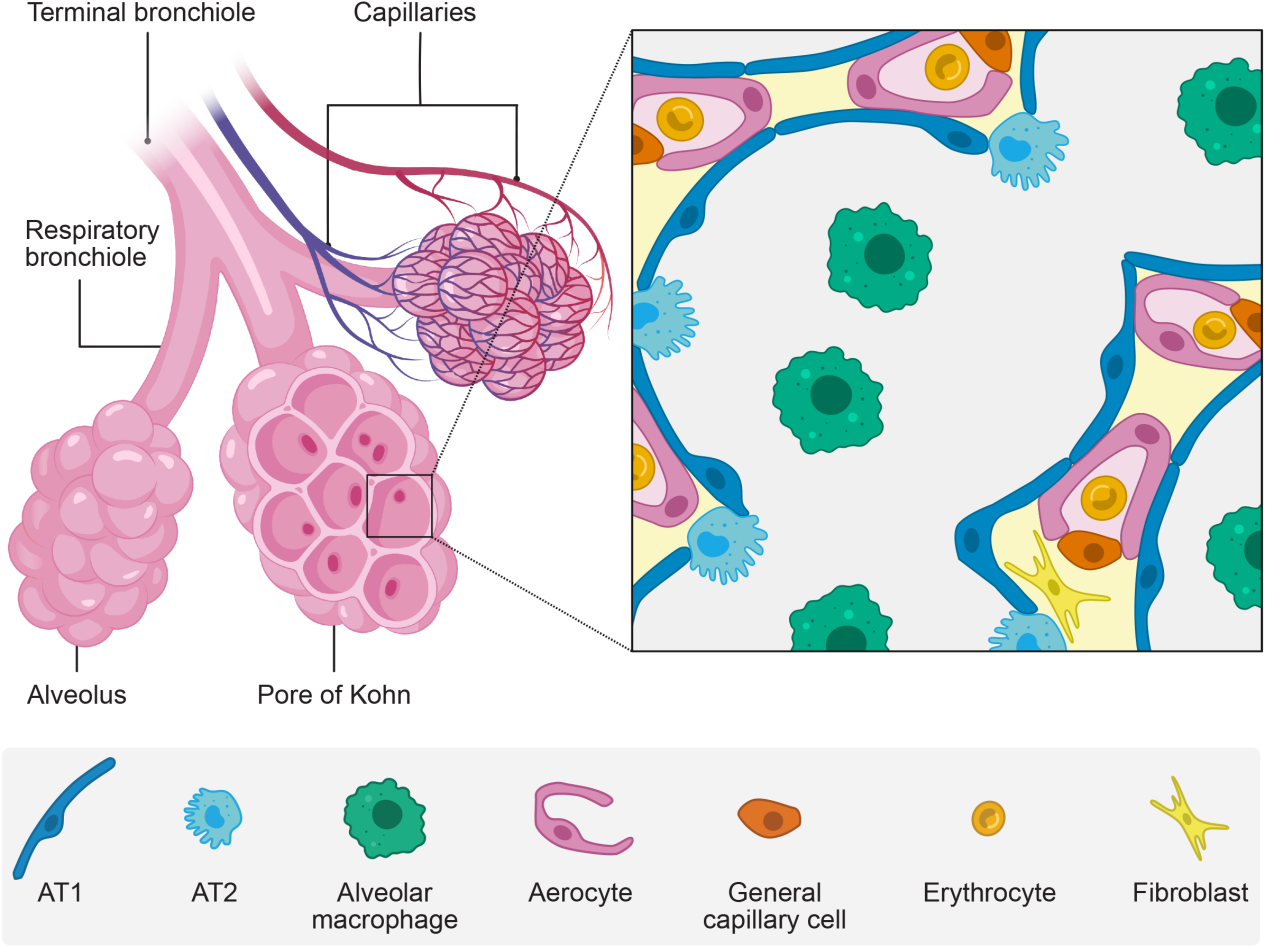
The alveoli. The alveoli are sac-like air compartments at the end of the bronchial tree with a unique epithelium specialized for gas exchange with the vascular system. The figure shows schematically a macroscopic view (left) and a microscopic view (zoom) of the alveoli. Created with BioRender.com and Affinity Designer.

The terminal bronchioles divide into several respiratory bronchioles, each ending in three or more sac-like alveoli. These alveoli are connected to one another and adjacent alveoli by pores of Kohn, enabling their common ventilation and preserving ventilation in case one of the terminal bronchioles is blocked (Kierszenbaum and Tres, 2019) (**Figure 1**). On average, alveoli have a surface area of 220,000 µm^2^ (Weibel, 2017). The partial air pressure is decreased to 104 mmHg within the alveoli, compared to 159 mmHg before inhalation at sea level, due to the increased temperature and humidity of the conducted air (Ortiz-Prado et al., 2019). During gas exchange, O_2_ diffuses from the alveolar lumen through the highly specialized epithelium and endothelium and attaches to red blood cells whereas CO_2_ diffuses in the opposite direction. The alveolar epithelium and endothelium form specialized adjacent layers (Weibel, 2017; Gillich et al., 2020).

The type 1 alveolar epithelial cells (AT1) (also called type 1 pneumocytes) lay the structural basis for efficient gas diffusion in the epithelium. These massively extending cells only have a few organelles and cover a mean surface area of 5,100 µm^2^, forming leaflets with a 0.1-0.4 µm thickness (Crapo et al., 1982; Weibel, 2017). AT1 cells form tight junctions with one another and type 2 alveolar epithelial cells (AT2) (also called type 2 pneumocytes) in a continuous epithelium (Weibel, 2017).

The alveolar epithelium is distinct from the rest of the respiratory epithelium. It is composed of unique cell types and is not strictly polar as AT1 and AT2 cell types can face more than one alveolar lumen (Weibel, 2017).

AT2 cells are bulky with apical microvilli. Despite being twice as frequent as AT1 cells, they only occupy 5% of the alveolar surface area (Crapo et al., 1982). They serve as the stem cells of the alveolar epithelium and regulate and coordinate the host’s defense and repair (Desai et al., 2014). Their cytoplasm includes large granular secretory bodies holding surfactant, an essential part of the thin liquid lining covering the alveoli’s air-directed surface (Knudsen and Ochs, 2018).

The alveolar liquid lining differs from the conducting airways’ thicker and viscous mucus layer. It forms a thinner film with two phases: a lower aqueous and an upper phospholipid phase (Weibel and Gil, 1968). The AT2 cells secreted surfactant consists of 90% lipids, predominantly phospholipids, and 10% proteins, including surfactant proteins (SP) (Knudsen and Ochs, 2018). SP-B and SP-C are essential to regulate the surface tension and are present in the upper phospholipid phase (Cochrane and Revak, 1991; Whitsett and Weaver, 2002). The hydrophilic antimicrobial SP-A and SP-D bind to the surface of different pathogens and facilitate their elimination by alveolar macrophages residing in the lumen and inhibit contact with epithelial cells (Watson et al., 2020). Among pathogens, the alveolar macrophages clear the alveolar lumen from inhaled particles, dead cells, and malformed surfactant (Shibata et al., 2001). Alveolar macrophages secrete proinflammatory cytokines and enhance pulmonary immune responses during infection (Jeyaseelan et al., 2005; Christenson et al., 2022). Furthermore, the epithelial cells secrete glycosaminoglycans (GAGs) which are part of the alveolar epithelial glycocalyx (Ochs et al., 2020). It is suggested that the glycocalyx and pulmonary surfactant components interact, enhancing each other’s functions (Lu et al., 2005).

## 3 Introduction into glycans

Glycans are fundamental building blocks of life, along with nucleic acids, proteins and lipids (Stroh and Stehle, 2014). Their high structural heterogeneity and functional versatility may be one of the reasons why they have been largely overlooked in research (Shivatare et al., 2022). Glycans serve various crucial roles that can be grouped into four main categories: Structural and modulatory functions, molecular mimicry of host glycans, and both intrinsic and extrinsic recognition (Varki and Lowe, 2009). Presented on the cell surface, glycans play a pivotal role in extrinsic recognition by interacting with factors such as pathogens.

Glycans can be categorized into glycoconjugates and GAGs. Glycoconjugates are glycans attached to a core protein or lipid, forming glycoproteins, proteoglycans, and glycolipids (Shivatare et al., 2022). Proteins can become glycosylated during post-translational modification, whereby glycans are attached. This process can result in either N-linked or O-linked glycans, depending on whether the glycan is attached to the protein through a nitrogen atom of an asparagine or an oxygen atom of a serine or threonine (Shivatare et al., 2022). Both glycoproteins and glycolipids can feature a terminal sialic acid at the end of the glycan chain. Sialic acids, a group of nine-carbon acidic amino sugars, are notably diverse, with α5-N-acetylneuraminic acid (Neu5Ac) being the most common. These sialic acids play a crucial role in external recognition (Weis et al., 1988).

GAGs are long, unbranched polysaccharides composed of repeating disaccharide units. Each unit consists of a hexuronic acid and a hexosamine. The hexuronic acid can be either D-glucuronic acid (GlcA) or L-iduronic acid (IdoA), while the hexosamine can be N-acetyl-glucosamine (GlcNAc) or N-acetyl-galactosamine (GalNAc). The glycosidic linkages between these units vary, resulting in different structural geometries (Gandhi and Mancera, 2008). GAGs are categorized into five main types based on their disaccharides and linkages: hyaluronic acid, chondroitin sulfate (CS), dermatan sulfate, heparin/heparan sulfate (HS) and keratan sulfate (KS). KS is structurally distinct because it contains D-galactose instead of a hexuronic acid (Gandhi and Mancera, 2008). GAGs can exist freely or be part of glycoconjugates, forming proteoglycans when bound to proteins. Hyaluronic acid is the only GAG that exists freely, while the others are typically conjugated to core proteins through a trisaccharide linkage. This linkage is composed of two D-galactose units and a D-xylose residue attached to a serine of the protein (Gandhi and Mancera, 2008). Most GAGs are sulfated, giving them a net negative charge and influencing their properties and interactions. Hyaluronic acid, however, is not sulfated but negatively charged (Gandhi and Mancera, 2008).

**Figure 2:**
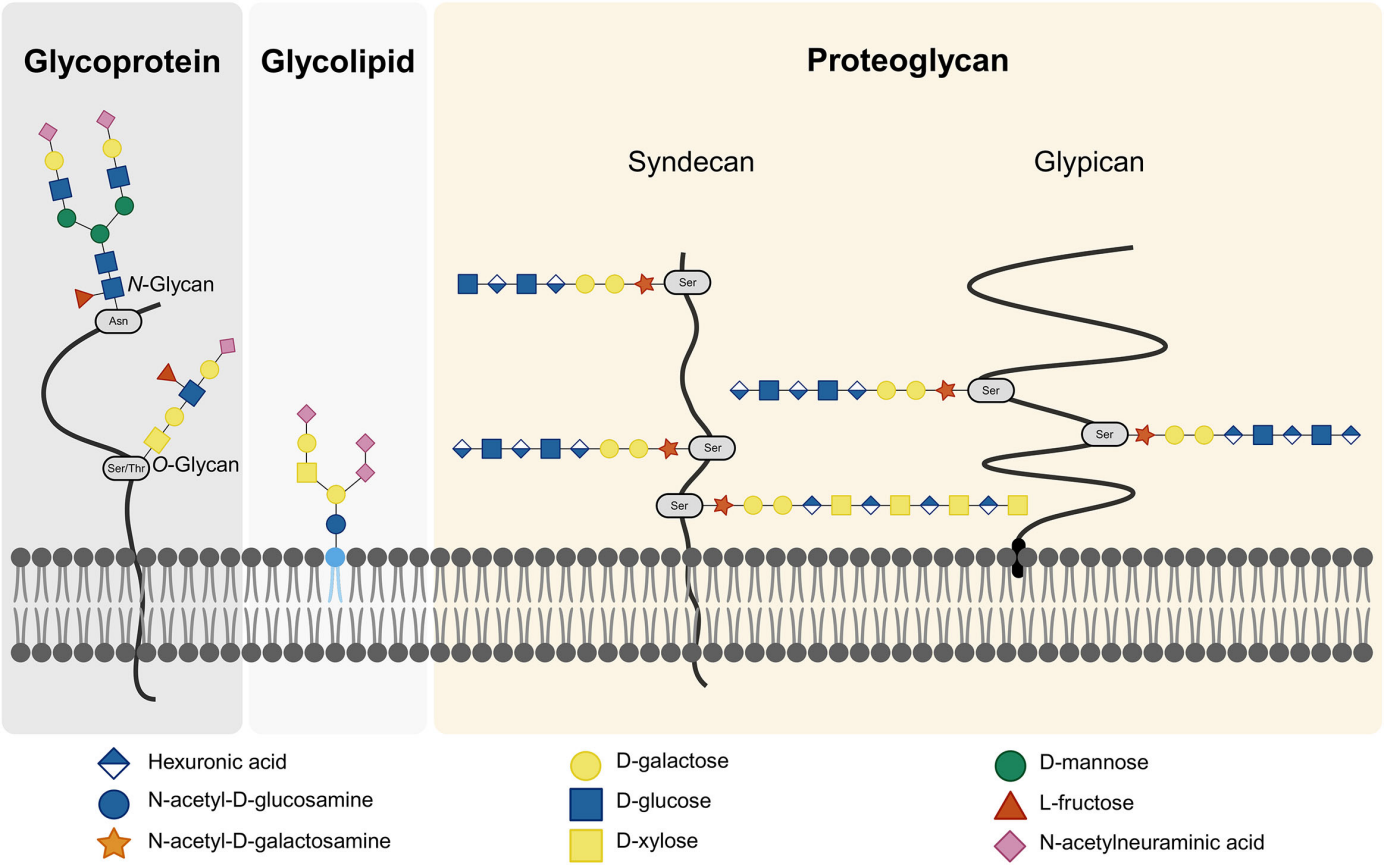
Cell Surface Glycans. The cell surface glycocalyx comprises glycoproteins, glycolipids, and proteoglycans. Glycoproteins can feature either N-linked or O-linked glycans, depending on the amino acid in the core protein to which the glycans are attached. Proteoglycans are proteins linked to GAGs. Within proteoglycans, a distinction is made between syndecans and glypicans based on the type of core protein. Created with BioRender.com.

CS (GlcA and GalNAc) and HS (GlcA and GlcNAc), the two most highly sulfated GAGs, occur on the cell surface. Cell surface GAGs are linked to core proteins which can be classified as either syndecans or glypicans. Syndecans are integral membrane proteins with a small cytoplasmic domain and a large extracellular domain. In contrast, glypicans are anchored to the plasma membrane via a C-terminal glycosylphosphatidyl-inositol (GPI) anchor and consist solely of an extracellular domain (Olofsson and Bergstrom, 2005) (**Figure 2**). The combined presence of glycoproteins, GAGs and proteoglycans constitutes the glycocalyx (Rizzo and Schmidt, 2023). The glycocalyx is a specialized extracellular matrix (ECM), closely interacting with the cell (Moore et al., 2021).

### 3.1 Glycocalyx of the alveolar epithelium

The glycocalyx is also found on the alveolar epithelium in the respiratory tract. The main components of this glycocalyx are HS, CS and hyaluronic acid (van Kuppvelt et al., 1985). Notably, hyaluronic acid is not directly bound to a core protein but is rather attached to receptors or integrated into the plasma membrane, leading to tissue hydration and viscosity (Ochs et al., 2020).

The alveolar epithelial glycocalyx is involved in viral infections of the respiratory tract, which means that the components of the glycocalyx can serve as initial attachment factors for viruses. These low-affinity interactions help concentrate virus particles on the cell surface, facilitating more specific interactions (Koehler et al., 2020). Depending on the virus, different glycoepitopes are recognized. For glycoproteins and glycolipids, terminal sialic acids are recognized whereas for GAGs, internal sequences are targeted (Olofsson and Bergstrom, 2005). Both glycoconjugates containing sialic acids and sulfated GAGs carry a negative charge, allowing them to interact with viral surface proteins through electrostatic interactions (Koehler et al., 2020). More specific interactions can involve hydrogen bonds and interactions between hydrophobic and aromatic amino acids of viral proteins and elements of the glycoepitopes (Koehler et al., 2020). For example, sialic acids comprise a high number of functional groups enabling them to form hydrogen bonds, salt bridges and non-polar interactions (Neu et al., 2011).

The alveolar epithelial glycocalyx can be degraded if, for example, the lung is injured. When this occurs, HS and CS are shed in an intact and highly sulfated form, suggesting cleavage of the anchoring proteoglycans (Rizzo and Schmidt, 2023). This degradation can potentially lead to pulmonary surfactant dysfunction and increased permeability of the alveolar epithelium (Rizzo and Schmidt, 2023). The phenomenon of GAG shedding has also been observed following influenza infection (Langouet-Astrie et al., 2022), and will be explored in more detail later.

## 4 IAV

IAV infections affect about one billion people annually (Mostafa, 2023). Often, an infection causes only mild symptoms. Still, each year, several million people exhibit severe symptoms, such as acute respiratory distress syndrome (ARDS), which is associated with alveolar barrier loss (Ware and Matthay, 2000). Thus, about 500,000 people die each year due to an IAV infection (Mostafa, 2023).

Influenza viruses are classified according to their stable nucleoprotein antigen into types A, B, C and D. All influenza viruses belong to the Orthomyxoviridae family. Accordingly, they possess a negative-sense, single-stranded, segmented RNA genome (Baltimore, 1971; Palese, 2001). IAVs are further subdivided into subtypes according to the combination of the surface glycoproteins hemagglutinin (H) and neuraminidase (N) (Noda, 2012).

The IAV viral genome is enveloped in cell membrane parts of its previous host cell and segmented into 8 parts. The segmented genome allows for random reassortment of viral segments in cells infected with the same type of influenza virus (Nakajima, 1997; Shimizu, 1997). This antigenic shift leads to the emergence of new pandemic strains, as there is usually no prior immunity to the new surface glycoprotein antigens (Shimizu, 1997; Treanor, 2015). In contrast, seasonal strains are caused by antigenic drift, which is the gradual mutation of genomic sequences, especially in the H and N gene segments, occurring over time due to selective pressure on the currently prevalent strain (Gething et al., 1980).

IAV circulation is not limited to humans, and there are huge animal reservoirs with foreign strains that could spill over to humans, originating from wild birds and finding their way to us through adaptations in domestic animals (Parrish et al., 2015). 16 H and 9 N genetically distinct variants have been identified in birds, ready for reassorting and increasing the diversity of natural reservoirs (Fouchier et al., 2005).

### 4.1 IAV-cell attachment

Viral attachment is initiated by recognition of terminally attached sialic acids, located on the host cell and mediated by the viral H glycoprotein. Portions of the head domain recognize either galactose α-2,3 or α-2,6 linked sialic acids attached to glycolipids or glycoproteins on the cell surface (Xiong et al., 2013; Eriksson et al., 2019). Avian strains prefer the α-2,3 linkage, as this is the most abundant sialic acid linkage in the avian intestinal infection site, while human strains prefer the α-2,6 linkage, which is most abundant in the human respiratory system (Matrosovich et al., 1997; França et al., 2013; Eriksson et al., 2019).

Until recently, the role of the glycocalyx during infection was almost overlooked. But further research showed that the degradation of the glycocalyx, also known as glycocalyx shedding, caused by viral infection can drive ARDS pathogenesis (Rizzo and Schmidt, 2023).

Glycocalyx shedding in mice aggravated the symptoms of ARDS by inducing surfactant dysfunction, and patients with more severe shedding required mechanical ventilation for longer periods of time (Rizzo et al., 2022). More specifically, glycocalyx degradation in mice following intratracheal lipopolysaccharide or bleomycin administration is thought to be initiated by cleavage of HS at the cell surface by metalloproteinase sheddases, resulting in increased lung permeability and impaired lung recovery (Haeger et al., 2018; LaRivière et al., 2020). The cleavage and shedding of HS also induces the shedding of CS and hyaluronic acid into the airspace. In one study, the glycocalyx degradation products were found to be shed and persist in the epithelial airspace for three weeks (LaRivière et al., 2020; Rizzo et al., 2022). Although glycocalyx shedding is highly heterogeneous in patients, with some patients experiencing severe glycocalyx shedding while others were barely affected, one review hypothesizes that shedding may be part of the defense mechanism against IAV infection by coating the approaching virions with glycocalyx components and thereby preventing viral cell entry (Rizzo et al., 2022; Hook and Bhattacharya, 2024). Nevertheless, as glycocalyx shedding has been correlated with more severe symptoms and a prolonged disease state, this hypothesis cannot be accepted at this time. The exact mechanism of glycocalyx shedding initiation remains to be resolved (Rizzo et al., 2022; Rizzo and Schmidt, 2023).

### 4.2 IAV subtype H5N1

Pandemics caused by genetic IAV reassortments, meaning all antigenic shifts and drifts that lead to the pandemic outcome, are not uncommon. In 2009, a swine variant of the still circulating H1N1 IAV caused the last human IAV pandemic (Smith et al., 2009). During the first year of this pandemic, there were 18,500 laboratory-confirmed deaths caused by this swine variant, but the estimated number of deaths associated with H1N1 is about 15 times higher and ranges between 151,700 and 575,900 (Dawood et al., 2012). In the past two and a half decades, a novel avian influenza strain has emerged as a significant public health concern with the potential to eventually cause the next pandemic in humans. In 1997, the first documented case of human infection with the influenza A subtype H5N1 was reported in China, following outbreaks on multiple poultry farms (Claas et al., 1998). Since 2003, 889 human infections have been reported from 23 countries, with nearly half of them proving fatal (Who, 2024). The virus has since spread among poultry through wild birds to multiple continents (Kandeil et al., 2023). Since 2020, clade 2.3.4.4b has expanded intercontinentally in birds throughout Europe, Asia, and Africa (Who, 2022). In 2021, the clade was first detected in North America, where it has undergone reassortment with North American avian influenza A genes, increasing the severity of infection in animal models (Caliendo et al., 2022; Kandeil et al., 2023). To date, the virus has been identified in wild birds, poultry, and dairy cattle herds in 12 different states of the US and transmission has likely occurred via lateral transfer from individuals introduced into new herds (Animal, Plant Health Inspection Service USDoA, 2024). A total of 13 human infections associated with clade 2.3.4.4b have been documented in the United States (Fao et al., 2024).

### 4.3 Necessary adaptations by H5N1 towards a major pandemic risk

In order for the virus to cross species and efficiently spread in humans, it must adapt from avian to mammalian preferences. The necessary adaptations are briefly summarized in **Table 1**.

**Table 1:** Comparison of human and avian IAV strain preferences.

Strain/ Property	Human	Avian
Epithelial cell preference	AT1	AT2
Place of infection	Respiratory system	Intestinal tract
Preferred sialic acid linkage	α-2,6	α-2,3
pH value of H activation	5.5 or below	5.6-5.9
Polymerase activity & replication efficiency	Human preferences	Avian preferences

To cross species barriers, avian strains have to adapt to human preferences. The main differences of infection preferences of human and avian IAV strains are listed below.

IAVs of different origins are known to target different AT cell types. Strains already circulating in humans, such as H1N1 and H3N2, are more likely to attach to AT1 cells while strains of avian origin, such as H5N1, H5N9 or H6N1, favor AT2 cells (Gu et al., 2007; van Riel et al., 2007). In birds, the viral infection originally takes place in the intestinal tract whereas in mammals, including humans, IAV infections affect the respiratory system (Eriksson et al., 2019). Since the most abundant sialic acid linkage on the cell surface is α-2,3 in the avian intestine and α-2,6 in the human respiratory system, it was thought that a switch in binding site preferences would be necessary to adapt from avian to human hosts (Matrosovich et al., 1997; França et al., 2013). This can be achieved by mutating only one amino acid in the nucleotide sequence of the H segment (Rogers et al., 1983; Shi et al., 2014). The transition from birds to humans was thought to span several host species, especially pigs, because their expression of both sialic acid linkages made them so-called “mixing vessels” for IAVs (Ito et al., 1998; Garten et al., 2009; Krammer et al., 2018). However, several recent studies have questioned the necessity for the virus to jump from birds to humans via pigs, suggesting the possibility of direct transmission instead (Eriksson et al., 2019; Liu et al., 2022b; Hook and Bhattacharya, 2024). The underlying explanation may be that SA*α*2,6Gal and SA*α*2,3Gal are differentially expressed in the respiratory system, which results in avian and human IAV viruses affecting different parts of the respiratory system (Shinya et al., 2006). SA*α*2,6Gal is highly expressed by cells forming the epithelium of the soft palate, nose, trachea, and bronchi (Shinya et al., 2006). SA*α*2,3Gal is most prominent at non-ciliated cells of the alveolar openings and on the surface of AT2 cells within the alveolar lumen (Shinya et al., 2006). As human strains most efficiently infect and proliferate in cells of the upper respiratory tract, the inflammation caused by the infection is mostly limited to this area (Shinya et al., 2006). The symptoms are often mild, and the infection usually ends within a couple of days without medical intervention (Guibas and Papadopoulos, 2017). Nonetheless, the efficient proliferation of the upper respiratory tract results in an effective human-to-human spread as nasal discharges contain high titers of live virus (Gralton et al., 2013).

On the other hand, avian strains such as H5N1 predominantly infect cells within the human respiratory zone and, therefore, often result in severe inflammation of the alveolar epithelium (pneumonia) which can be lethal. However, without efficient proliferation in the upper respiratory tract, human-to-human transmission is unlikely (Shinya et al., 2006). As noted previously, inhaled air and particles must pass through the upper and lower conducting airways, which are designed to effectively trap and eliminate threats. IAV virions are present in liquid particles with a diameter smaller than 2 μm (Gralton et al., 2013). Inhalation studies in human lungs have confirmed that these particles are small enough to reach the alveolar lumen, given the correct breathing pattern, thereby bypassing the conducting epithelium (Heyder et al., 1986). Within the alveoli, the alveolar liquid lining layer and macrophages are the last line of defense in inhibiting the viral attachment and infection of AT2 cells (Tumpey et al., 2005; Hook and Bhattacharya, 2024). In addition to AT2 cells, viral replication of H5N1 was previously not only found in the trachea but the infection also spread to other organs including the brain and to a fetus through the placenta in three post-mortem tissue samples (Gu et al., 2007). The more severe courses of infection caused by avian IAV strains are hypothesized to be due to the preferred AT2 cells being twice as numerous in the alveolar epithelium than the AT1 cells preferred by human strains (van Riel et al., 2007). This is supported by ARDS in H5N1 infected macaques being mainly driven by excessive viral replication rather than cytokine storms (Wonderlich et al., 2017).

After viral endocytosis into the host cell, the virus-containing endosome is acidified. This triggers a conformational change in the H surface protein, which initiates the release of the viral genome into the host cell for translation and replication (Bullough et al., 1994). The exact pH value at which H activation occurs varies between avian and human strains. In avian IAV strains, activation of H occurs at higher pH values, closer to 6.0 (5.6-5.9), with the highest mortality in chickens being reached at pH values of activation of 5.7 (Reed et al., 2010; DuBois et al., 2011). H proteins of human IAV strains are activated at lower pH values than avian strains, with membrane fusion being initiated at pH 5.5 or below (Russell, 2021). Finally, for efficient replication, avian IAV strains have to acquire some mutations in their polymerase segments that improve polymerase activity and replication efficiency in human cells, as has been seen in other viral adaptations (Subbarao et al., 1993; Wang et al., 2013).

### 4.4 Adaptations of clade 2.3.4.4b isolates

A 2023 study evaluated the transmission potential of H5N1 clade 2.3.4.4b isolates in chickens and ferrets. Lateral transmission among chickens was observed but no transmission to ferrets occurred, and the viruses were unsuccessful in spreading from ferret to ferret. However, the eagle isolate, which had already undergone reassortment with North American gene segments and gained polymerase and nucleoprotein genes, resulted in more severe diseases and higher viral loads in ferrets than the wigeon isolate without North American gene segments. In addition, the eagle isolate was also found in brain tissue three days after infection, and ferrets inoculated with this isolate exhibited severe neurological symptoms (Kandeil et al., 2023). This is consistent with previous observations in humans where virus infiltration into brain tissue was demonstrated and neurotropism was confirmed (Gu et al., 2007; Rajabali et al., 2015). In summary, the more North American gene segments acquired by different virus isolates, the more severe the disease outcome in ferrets. In addition, all isolates that underwent reassortment with North American wild bird IAVs showed systemic viral spread and led to neurological symptoms. In the same study, these results were replicated in mice (Kandeil et al., 2023). With respect to human host adaptation, the wigeon and eagle virus isolates were tested for their preferred sialic acid binding, H activation pH value, and viral replication rates. Both isolates favored binding to the avian preferred α-2,3-linked sialic acids and their H activation pH value was 5.8, within the avian range (Kandeil et al., 2023). Only replication rates in undifferentiated Calu-3 cells and primary differentiated human airway cultures were different, suggesting changes within the polymerase segments. Finally, the use of N inhibitors has been shown to still be effective in stopping viral replication in all tested isolates (Kandeil et al., 2023). For now, transmission to humans and lateral transmission between humans remain unlikely, especially without the property to replicate in the nose and trachea, until further adaptive mutations are acquired, but the potential threat of this IAV strain is being closely monitored (Fao et al., 2024).

## 5 3D lung cell culture systems

Researchers are progressively diverging from *in vivo* experiments and are seeking alternative options that precisely depict the processes occurring within living organisms while confirming the observations made in normal two-dimensional (2D) cell culture. Thus, novel *in vitro* three-dimensional (3D) cell culturing systems are emerging. The need for the development of these culturing systems is driven by the ethics of animal testing but also by the lack of complexity in normal 2D cell culture (Hoarau-Véchot et al., 2018) as well as by the translational differences between animal and human data (Dichtl et al., 2024).

### 5.1 2D *vs*. 3D cell culture

While 2D cell culture systems grow on an adherent surface as a monolayer (Kapałczyńska et al., 2018), 3D cell systems have the ability to grow and interact with their surroundings in all three dimensions (Urzì et al., 2023). These systems mimic the physiological conditions that cells experience *in vivo*, taking into account the additional dimension of the cell-cell, cell-extracellular matrix, and cell-environment interaction that single cells experience once a multicellular tissue emerges. As these interactions occur in 3D cell systems, the cellular response, morphology, and physiology are affected (Edmondson et al., 2014). These properties are not captured in traditional 2D cultivation techniques (Edmondson et al., 2014). Moreover, 3D cell systems also create a nutrient gradient, as observed *in vivo*, while nutrient availability is homogenous in 2D cell culture (Urzì et al., 2023). Additionally, 3D cell cultures enable cell propagation without requiring immortalization and provide more reliable predictions of drug responses in living organisms (Urzì et al., 2023), offering substantial advantages for translational research.

### 5.2 Advantages and limitations

Both 2D and 3D cell cultures have their respective advantages and disadvantages. The advantages of 2D cell culture over 3D cell culture are primarily economic: 2D cell culture is a well-established technique with a vast body of research and it is relatively simple to observe and characterize cells grown in 2D. In contrast, 3D cell culture can be expensive, depending on the cultivation method used, and it can be challenging to find the appropriate analysis assay. Additionally, reproducibility can be a concern in 3D cell culture (Urzì et al., 2023).

3D cell culture systems, however, offer several advantages over both 2D systems and animal models. Beyond addressing the ethical concerns associated with animal testing, these systems allow for more favorable control of variables, more accurate translation of *in vitro* results to humans and a reduction in maintenance costs (Bédard et al., 2020).

### 5.3 Selection criteria for 3D cell culture systems

The choice of the 3D cell system depends on the specific purpose and functions one wants to study, as not all cultivation systems achieve the same properties or can be used to study viral infections as seen in extensive reviews discussing *in vitro* lung models (Plebani et al., 2022; Shah et al., 2023; Dichtl et al., 2024).

Selection criteria include the choice of cells (single or multiple cell types depending on the required complexity), available fabrication and incubation technology, required incubation time, and economic considerations. **Table 2** provides a comprehensive overview of the advantages and disadvantages of different 3D cell culture systems to guide researchers in making appropriate selections.

**Table 2:** Summary of the advantages and disadvantages of the discussed 3D cell culture systems.

System	Advantages	Disadvantages
3D bioprinted models	· controlled design · high-resolution	· investment of time and material · mechanical stress
Lung-on-chip	· biochemical cues · recreation of different regions	· fabrication is exclusive · low throughput
Spheroids	· self-assembly · simplicity and speed	· lack of complexity
Organoids	· developmental stages · different cell types · *in vivo* morphology	· inconsistent organization · nutrient diffusion limitation · time-consuming establishment
ALI	· *in vivo* morphology · co-culture	· unwanted cell-membrane interactions · physical factors need to be considered
ALI-OTE	· incorporation of sECM · hydrogel · *in vivo* morphology	· Lacks epithelium-endothelium interaction · Isolation of sECM

In the following section, we summarize the different approaches and criteria to consider when choosing cultivation technique of 3D lung cell culture systems, especially in the context of viral pulmonary infections. Ultimately, we propose a novel cultivation technique based on the model developed by Leach and colleagues in 2023 (Leach et al., 2023) that can be employed to investigate IAV infection in the lung and ascertain the role of GAGs in viral infection.

### 5.4 Scaffold-based 3D cell culture models

The different 3D cell culture techniques can be grouped into two categories, scaffold-based and scaffold-free cultivation techniques (Edmondson et al., 2014).

Scaffold based techniques are techniques where the 3D cell culture system relies on solid support, i.e. a scaffold, that consists of a porous matrix (Alghuwainem et al., 2019). An example of a porous matrix would be a biocompatible hydrogel, a polymer that can be swollen in water, such as collagen, alginate, or hyaluronic acid (Caliari and Burdick, 2016). The choice of hydrogel also depends on the properties one wants to achieve as there are naturally occurring hydrogels (i.e. collagen and alginate) or synthetic hydrogels (i.e. polyacrylamide and polyethylene glycol). An excellent and detailed review by Caliarli and Burdick has been published that deals with the hydrogel selection depending on the experimental requirements (Caliari and Burdick, 2016).

#### 5.4.1 3D bioprinted models

3D bioprinted models represent an advanced scaffold-based approach that uses computer-aided designs in order to create the complex lung architecture (Ni et al., 2022). The creation of the systems can be controlled in a layer-by-layer manner, based on the choice of the 3D printing system, by mixing the cells with a hydrogel or adding the cells after depositing the hydrogel. The printing process enables the creation of high-resolution and functional models that closely resemble the *in vivo* morphology. This is achieved by taking into account the inherent cell heterogeneity, the composition of the ECM, and the complex organization that can be achieved through bioprinting (de Melo et al., 2021). The cells are viable, can function, and have an increased migratory capability in the printed model (Ni et al., 2022).

Bioprinted lung 3D models have seen applications in various cases. Berg and colleagues developed an optimized hydrogel and 3D-printed lung tissue consisting of human alveolar epithelial cells to investigate IAV infection (Berg et al., 2018), while Lee et al. developed a three-layered bioprinted airway model to study severe-acute-respiratory-syndrome coronavirus 2 (SARS-CoV2) infection by using vascular endothelial cells, collagen-based extracellular matrix and human airway epithelial cells. The bioprinted model was then cultured at the ALI to mimic the airway environment of the lung (Lee et al., 2024). However, this method necessitates the use of a 3D bioprinter, thereby rendering it less straightforward than other methods and requiring a significant investment of time and materials, which can be costly and of limited accessibility. The printing process itself introduces mechanical stress, typically in the form of shear stress (Ni et al., 2022), to the cells which can decrease their viability.

#### 5.4.2 Organ-on-chip models

Another scaffold-based model that has attracted a lot of interest is the organ-on-chip model that combines the research area of microfluidics and bioengineering (Ni et al., 2022), with lung-on-chips being a prominent representation of those models. The basic architecture of lung-on-chips models involves two microchannels, upper and lower, separated by a thin polymer membrane (Ni et al., 2022). First generation lung-on-chips use polydimethylsiloxane (PDMS) as a separation membrane, while second generation chips are moving further away from synthetic polymers and are heading instead towards natural hydrogel membranes with ECM components such as collagen-elastin membranes (Zamprogno et al., 2021).

Lung-on-chips are often used to mimic the air-blood interface by culturing primary lung alveolar epithelial cells in one compartment and primary lung endothelial cells in the other compartment. An alternative approach to lung-on-chip models involves the separation of epithelial and endothelial layers, thereby creating an artificial small airway-on-a-chip. This is achieved by culturing primary human airway bronchiolar epithelial cells on a porous membrane within one microchannel, while lung microvascular endothelial cells are cultured on the opposite side of the same membrane in the second channel. This process results in the formation of a mucociliated epithelium–endothelium, that mimics the natural mucosal safety barrier in the lung (Benam et al., 2017). This would constitute an ideal surface layer for the interaction of synthetic H5N1 viruses, mimicking the structure of the human lung.

As *in vivo* morphology plays an important role in developing 3D cell culture models, Baptista and colleagues developed a lung-on-chip model that recreates the spherical geometry of the alveolar region of the lung by using a micro-curved porous membrane (Baptista et al., 2022). Since these models can be also cultured by exposing the cells to air, an ALI can be formed. However, because the cultivation happens in a confined chamber it requires special equipment to fabricate the chips or even special access to facilities. The lung-on-a-chip also yields low throughput as it depends on the size of the chip (Ni et al., 2022).

### 5.5 Scaffold-free 3D cell culture models

The second category of 3D cell culture systems are scaffold-free models. Scaffold-free techniques, in comparison to scaffold-based models, do not rely on the solid support of porous matrices and depend on the inherent ability of the desired cells to aggregate without external support, and create a 3D environment on their own without external cues. Once cultivated, those cells can self-assemble and create 3D models (Langhans, 2018).

#### 5.5.1 Spheroid models

Spheroids are one of the easiest scaffold-free 3D cell culture systems, first gaining popularity in cancer research (Kapałczyńska et al., 2018). They can be used, for example, to mimic alveolar cells (Ni et al., 2022) or human bronchial epithelial cells (Baarsma et al., 2022). This method relies solely on the aggregation properties of the cell on a non-adhesive surface, mostly in suspension (Ni et al., 2022). The generation of spheroids can be achieved through the utilization of a single cell type or a combination of multiple cell types. Researchers tend to gravitate towards this method as the production of the spheroids is a fast process. However, since this system is very simple, it lacks complexity and mimics only a small part of the organ, thus, not forming the exact *in vivo* morphology (Paolicelli et al., 2019).

#### 5.5.2 Organoid models

Another scaffold-free 3D cell culture system is “mini-organs”, better known as organoids. Organoids are multi-cellular aggregates formed by stem cells or airway epithelial progenitor cells such as alveolar cell type II (Ni et al., 2022), that can self-organize and differentiate into functional cell types that mimic *in vivo* morphology of the organ displaying essential features (Paolicelli et al., 2019). They can mimic the different developmental stages as seen *in vivo*, while also being used to study the cell-cell or cell-matrix interactions (Ni et al., 2022). A lung organoid cell culture system has been developed by Zhou and colleagues, that contain ciliated, goblet, club, and basal airway epithelial cells used to study the infectivity of emerging IAV strains (Zhou et al., 2018). However, establishing an organoid cell culture for an accurate representation of the lung can be time consuming, as the co-culturing of different cell types tends to be difficult (Ni et al., 2022) and lacks consistent cellular organization (Hofer and Lutolf, 2021). Due to the restrictions of nutrients and diffusion, size and cell death are limiting factors, thus the growth of the organoid cannot be controlled (Hofer and Lutolf, 2021).

### 5.6 Air-liquid interface models

**Figure 3:**
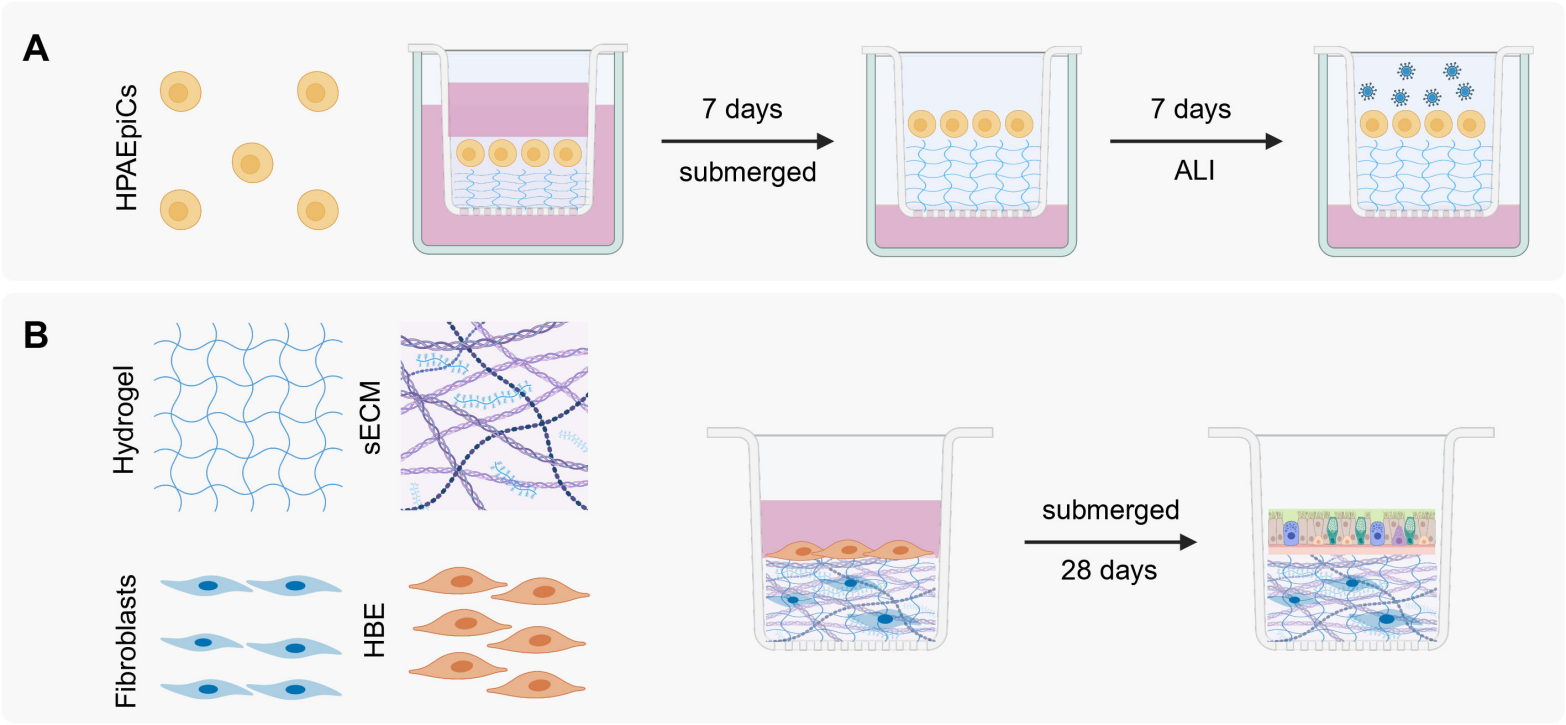
ALI cell culture models. **(A)** ALI scaffold-based 3D cell system, adapted from Bhowmick et al (Bhowmick et al., 2018). HPAEpiC: human pulmonary alveolar epithelial cells. After 7 days at ALI and the formation of a monolayer, viruses can be added to study the infection cycle. **(B)** ALI OTE 3D cell culture system taken from Leach et al (Leach et al., 2023). HBE: human bronchial epithelial cells. After crosslinking the hydrogel the human bronchial cells are added and are submerged in medium for 28 days, allowing differentiation. Created with Biorender.com.

A prominent example of a 3D cell culture system that is employed frequently for modelling the lung is the ALI cell culture. This system can be classified as either scaffold-based or scaffold-free, depending on whether a membrane has been utilized for cultivation. At the interface of the external and internal environment lie the airways that consist of various epithelial cell types as mentioned in the section discussing the respiratory system. They perform various tasks, mainly clearance of mucus, keeping the airways at the right humidity, sensing of pathogen/particles, but also the response, and signaling to the underlying mesenchyme and immune system (Leach et al., 2023). Furthermore, the subepithelial ECM plays a crucial role in preserving the structure and function of the airways, as they promote proliferation, differentiation, and activation of the cells (Leach et al., 2023). To study these complex systems, ALI cell culture systems have been developed since 1988 by Whitcutt and colleagues (Whitcutt et al., 1988) and have seen major improvements ever since. The ALI cell culture system follows the basic structure as seen in **Figure 3A**.

The ALI cell culture is primarily comprised of an external plastic culture dish and an internal porous membrane insert, where the desired cells are cultured on the matrix insert (eg. Collagen-Chitosan) and left to grow to confluency while submerged in medium. After the medium is removed, it can be observed that the cells start forming a uniform layer, having an elongated/squamous and spherical form as seen *in vivo*, where it consists of alveolar type I and II cells (Bhowmick et al., 2018). An important observation and advantage is that the cells start forming a mucosal layer, which consists of water, ions, GAGs (eg. KS, HS, and hyaluronic acid), and mucins (Iverson et al., 2020). This layer is important for gas exchange, lubrication, clearance of pathogens and a creation of a barrier (Fahy and Dickey, 2010). The model can even be improved by adding a layer of hydrogel that can mimic the stiffness of the lung, providing this model with the mechanical cues that are observed *in vivo* (Bhowmick et al., 2018). The cells can then be infected with viruses to closely study the infection cycle closely as done by Bhowmick and colleagues (Bhowmick et al., 2018).

In a scaffold-free ALI the cells would be directly cultured on plastic dishes that are coated with collagen, without a hydrogel that supports the formed monolayers (Ni et al., 2022).

### 5.7 Air liquid interface organ tissue equivalent 3D cell culture model

The characteristics of both scaffold-based and non-scaffold-based models can be combined to develop a model that incorporates the most advantageous aspects of each. Leach and colleagues introduced a novel 3D cell culture model known as the air-liquid interface airway organ tissue equivalent (ALI-OTE). This system integrates aspects of both ALI cell cultures and scaffold-based models by growing human bronchial epithelial cells on a hydrogel layer embedded with lung fibroblasts and sECM components (**Figure 3B**) (Leach et al., 2023).

The ECM elements include collagen, elastin, laminin, fibronectin, sulfated GAGs, and hyaluronic acid whereas the hydrogel itself is composed of thiolated gelatin, hyaluronic acid, and a polyethylene glycol crosslinker to reduce hydrogel contraction. Following mixing, the hydrogel undergoes UV crosslinking before a second cell type is introduced while submerged in medium. The differentiation process spans 28 days. This model offers flexibility as it can be cultivated on transwells or within microfluidic chips.

A key advantage of this model is the incorporation of sulfated GAGs into the hydrogel, allowing for the study of GAG function in relation to IAV infection. Additionally, the inclusion of native lung fibroblasts supports cell attachment and basement membrane formation as fibroblasts are known to secrete ECM components within the hydrogel (Leach et al., 2023). The thiolated modification of the hydrogel backbone minimizes hydrogel contraction, while the use of hyaluronic acid mimics the *in vivo* environment (Leach et al., 2023). Physical and mechanical cues are provided by the chosen hydrogel such as stiffness which influences the epithelial layer thickness. Furthermore, the incorporation of sECM into the hydrogel was achieved without altering the ECM content, thus allowing for the possibility of biochemical adjustments to the hydrogel if necessary (Leach et al., 2023).

The addition of the solubilized ECM components is crucial for the formation of the monolayer and providing the biochemical cues and interactions that are seen *in vivo* for instance epithelial attachment as well as differentiation. As the sECM contains GAGs, their role in viral interaction can be further studied. Substituting human bronchial epithelial cells with primary human pulmonary alveolar epithelial cells (HPAEpiC) in ALI culture systems for IAV (Bhowmick et al., 2018; Yang et al., 2022) studies should not cause any issues. Existing methodologies for model fabrication (Leach et al., 2023) and HPAEpiC maintenance (Yang et al., 2022) can be utilized, with the modified IAV added to study the infection cycle.

Additionally, alveolar cells produce sialic acid (Brandt et al., 2021) as the IAV binds to sialic acid on the host cell (Liu et al., 2022a). Therefore, this model can also help study this interaction further. Alveolar macrophages, as mentioned previously, are found in the alveolar space (Bissonnette et al., 2020) and play an important role in the infection cycle. They can be added after the differentiation of the HPAEpiCs but before the infection with the modified IAV to increase the complexity of the system.

Accurate *in vitro* alveoli models also require tunable air pressure, reflecting the 149 mmHg (Ortiz-Prado et al., 2019) partial pressure of oxygen in alveoli using cell pressure chambers. Those chambers have been widely used in various research aspects to accurately capture *in vivo* conditions (Nagamatsu et al., 2019).

While this novel ALI-OTE provides numerous advantages, it lacks the interaction between the epithelium and the endothelium. However, a modification of the model by Leach and colleagues to include endothelial cells should be feasible as the model utilizes a porous membrane. The epithelial cells can be cultured on the other side of the membrane, as seen in lung-on-chip models. A similar approach has been done by Licciardello and colleagues, where they first co-cultured alveolar epithelial and lung microvascular endothelial cells at ALI to promote AT I and AT II differentiation. Subsequently, lung fibroblasts incorporated into a collagen hydrogel were introduced, forming a tri-culture (Licciardello et al., 2023). This opens the room for an approach that harnesses the possibility of a tri-culture as seen by Licciardello and colleagues and the established model by Leach et al (Leach et al., 2023).

This OTE model was employed by another group to study gene expression alterations in response to viral infections including IAV. Following infection, changes in gene expression were identified and interferon-stimulated genes were upregulated. In addition, cellular structures changed, indicating altered priorities during infection, and 92% mean accuracy was achieved when attempting to classify respiratory viral infections using multinomial logistic regression. This study provided insight into the host defense mechanism and identified biomarkers of viral infection (Rezapour et al., 2024).

To summarize, incorporating sECM, native lung fibroblasts, and hyaluronic acid hydrogel creates an *in vitro* environment that closely resembles *in vivo* conditions and is suitable created for studying viral infections.

## 6 Synthetic virus models

To mitigate the risks of sporadic human infections when working with H5N1, the Centers for Disease Control and Prevention (CDC) issued recommendations in 2013 mandating that work with H5N1 be conducted in biosafety level 3 (BSL-3) laboratories (Gangadharan et al., 2013). These facilities incorporate stringent safety measures, such as air handling systems, mandatory showers, and restricted access protocols. However, the operational demands and resource-intensive requirements of BSL-3 laboratories pose significant barriers for conducting the extensive experiments needed to study H5N1.

In contrast, less pathogenic IAV strains can often be studied in BSL-2 laboratories, which have less restrictive requirements (Eisfeld et al., 2014). While this option facilitates research, it does not fully address the need for direct studies of the H5N1 strain.

An alternative approach involves the use of synthetic models that mimic the key characteristics of naturally occurring IAV strains without posing a threat to human health. These models recreate critical viral structures essential for studying cellular interactions and disease mechanisms but are incapable of causing infection. As a result, research can be conducted in BSL-1 or BSL-2 laboratories, significantly lowering logistical and safety barriers (Staufer et al., 2022). Another advantage of using synthetic virus models to study the interaction is the precise control over composition and biophysical properties (Staufer et al., 2022). This control enables a step-by-step study of the different viral replication cycle steps.

The following section outlines prominent examples of these synthetic virus models, which offer valuable tools for investigating IAV infections, particularly in the lung, and exploring the role of GAGs in pathogenesis.

### 6.1 Gold nanoparticle-based models

Gold nanoparticles (AuNPs) can be classified according to their dimensionality into one-dimensional shapes such as nanorods and nanotubes, two-dimensional structures like nanoplates, and three-dimensional forms including gold nanotadpoles (Elahi et al., 2018). Among these, nanorods are considered to be particularly suitable for mimicking viruses due to their dimensions, with diameters around 10 nm and lengths up to 100 nm, matching those of many natural viruses (Murphy et al., 2019).

AuNPs are synthesized using either top-down or bottom-up approaches (Elahi et al., 2018). Top-down methods, which are primarily physical, involve processes like laser ablation or ultraviolet radiation to break down bulk material into nanoparticles. In contrast, bottom-up approaches typically use chemical methods such as colloidal synthesis where solvated gold salts are reduced in the presence of surface-capping ligands. These ligands prevent particle aggregation through electrostatic or physical repulsion (Dreaden et al., 2012). A widely used synthesis method involves reducing an aqueous solution of chloroauric acid with citrate (Dreaden et al., 2012). To enhance biocompatibility and establish specificity, functionalization of the AuNPs is necessary. This can be achieved through chemical tuning of the surface or simple physical interactions such as hydrophobic-hydrophobic interactions (Dreaden et al., 2012). Gold surfaces also allow for functionalization using diverse anchoring groups, including thiolates, amines, and carboxylates, enabling tailored applications (Dreaden et al., 2012).

**Figure 4:**
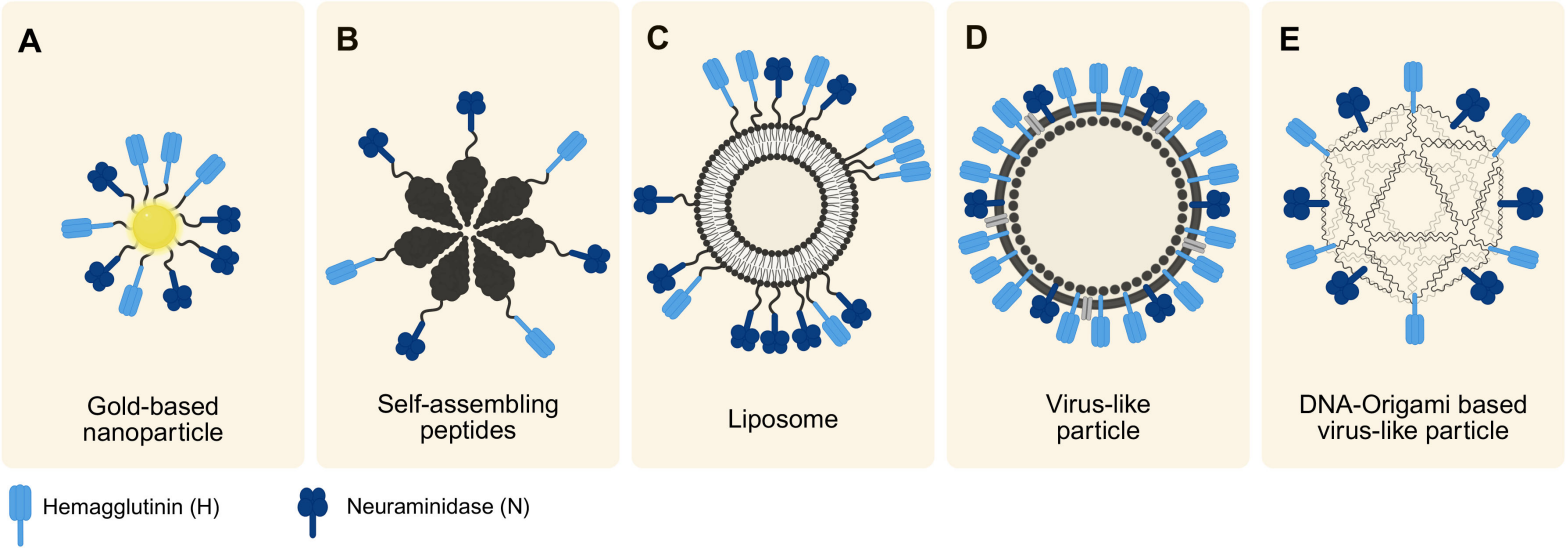
Overview of the discussed synthetic virus models. The models differ in their scaffolds but all incorporate IAV-relevant viral proteins (H and N), varying in their degree of order. Arrangement follows a trend from smallest to largest model from left to right. **(A)** Illustration of a gold-based nanoparticle typically within the size range of 10–30 nm. **(B)** Synthetic virus model created using self-assembling peptides ranging 30–50 nm. **(C)** Liposome-based model (size 20 nm-10 µm) with protein-enriched domains. **(D)** Typical design of a VLP ranging from 20–200 nm in size, showing a high order of hybridized proteins. **(E)** Schematic of a novel approach of using DNA-origami based virus-like particles (around 40 nm). Created with Biorender.com.

AuNPs (**Figure 4A**) have been utilized to study early infection of SARS-CoV-2 in an *in vitro* 3D upper airway model (Pennarossa et al., 2021). Their size and shape match those of the virus, making AuNPs suitable for such studies even without additional surface modifications. Depending on their size and functionalization, AuNPs serve a variety of additional applications. They can be conjugated with therapeutics for drug delivery or functionalized with targeting agents such as antibodies to enable targeted delivery (Daraee et al., 2016). Furthermore, their unique optical properties make them valuable for diagnostics, applying them in imaging such as confocal laser microscopy (Daraee et al., 2016).

### 6.2 Self-assembling peptides-based models

Self-assembling peptides (SAPs) have the ability to organize into structured forms without external intervention (Whitesides and Grzybowski, 2002). In nature, viral capsids are able to self-assemble through a complex and highly regulated oligomerization process (Mateu, 2013). Thus, SAPs are a useful tool to mimic viral capsids and their symmetries (Jankovic et al., 2021).

In materials science, SAPs can form a variety of nanoarchitectures, including nanotubes, nanovesicles, and nanofibers which are responsive to changes in environmental conditions such as pH, temperature, or ionic strength (Eskandari et al., 2017). These constructs offer significant advantages over naturally occurring peptides which are often unstable and prone to enzymatic degradation (Qi et al., 2018). The stability and functionality of SAPs stem from noncovalent interactions such as ionic bonding, hydrophobic forces, hydrogen bonding, and π–π stacking (Qi et al., 2018). The amino acid composition is a key determinant of assembly behavior as factors like charge, hydrophobicity, size, and polarity influence the resulting supramolecular structures (Eskandari et al., 2017). Consequently, the primary and secondary structures of the peptides dictate the self-assembly process (Negahdaripour et al., 2017) offering a level of tunability that enables the design of SAP-based models with controllable features.

The chemical synthesis of SAP monomers is typically achieved through solid phase peptide synthesis (SPPS) (Qi et al., 2018). Furthermore, they can be recombinantly expressed in host cell organisms (Negahdaripour et al., 2017). In vaccine development, a SAP platform and viral epitopes can be synthesized separately and then covalently linked to form immunogenic structures (Negahdaripour et al., 2017) (**Figure 4B**). Furthermore, they can be chemically or biologically produced together (Negahdaripour et al., 2017). For example, SAP monomers of the antigens M2e and Helix C can assemble into SAP nanoparticles to develop influenza vaccines (Karch et al., 2017). Beyond vaccine development, SAPs have broad applications in fields such as imaging, drug delivery, and regenerative medicine (Qi et al., 2018).

### 6.3 Liposome-based models

Liposomes are spherical vesicles composed of one or more phospholipid bilayers which can encapsulate aqueous solutions (Akbarzadeh et al., 2013). They can range in size from 20 nm to over 10 µm, existing as either unilamellar or multilamellar structures (Gao et al., 2018). Their lipid bilayer composition is often made up of phospholipids such as phosphatidylcholine, phosphatidylserine, and cholesterol (Gao et al., 2018).

Liposomes are commonly synthesized using methods like lipid film hydration or electroformation, with extrusion techniques often applied to refine vesicle size (Gao et al., 2018; Filipczak et al., 2020). Functionalization methods include the use of Ni-chelating lipids and protein conjugation through chemical techniques such as maleimide chemistry, enabling highly customizable designs (Wholey et al., 2020). Thus, viral antigens can be recombinantly expressed and then conjugated to the liposome (Wholey et al., 2020) (**Figure 4C**).

In influenza virus mimicry, pseudopeptidic polymers can replace viral spikes to provide a safer alternative to naturally derived peptides (Chen and Chen, 2016). In this approach, the liposome is composed of a phosphatidylcholine and cholesterol mix and is functionalized with a polyamide backbone containing the hydrophobic amino acid L-phenylalanine. This modification introduces pH-responsive characteristics that mimic the endosomolytic behavior of viruses. Due to their non-immunogenic nature, these liposomes serve as valuable tools for drug delivery (Chen and Chen, 2016) and are used in immunology, for vaccine development (Schwendener, 2014).

### 6.4 Virus-like particles

VLPs are nanoscale structures formed by the self-assembly of viral proteins, closely resembling actual viruses in shape but lacking genetic material, which makes them non-infectious (Nooraei et al., 2021). These particles typically range from 20 to 200 nm in size and can exhibit various structural forms such as icosahedral or rod-shaped configurations. Due to their strong structural similarity with real viruses, VLPs share visual and functional characteristics with them as seen in **Figure 4D**, yet they cannot replicate since they do not contain genetic material (Nooraei et al., 2021; Gupta et al., 2023).

VLPs can be categorized based on their lipid composition into two main types: enveloped and non-enveloped. Enveloped VLPs possess lipid membranes, whereas non-enveloped VLPs do not. Additionally, they are further classified as homologous or heterologous based on their protein composition. Homologous VLPs consist solely of proteins from their native virus, whereas heterologous VLPs incorporate proteins from diverse sources, which can enhance their immunogenic potential (Gupta et al., 2023) as the epitope diversity is increased.

The production of VLPs involves cloning the structural genes of the virus of interest, followed by the expression of self-assembling viral proteins in various systems. These systems range from prokaryotic hosts like bacteria and yeast to eukaryotic platforms such as baculovirus/insect cells, mammalian cells, and plants (Nooraei et al., 2021). For instance, H5N1 influenza VLPs can be produced using recombinant baculoviruses engineered to express hemagglutinin (H) and matrix protein (M1) genes. These genes, optimized for insect cell expression, are cloned into a plasmid, transformed into Escherichia coli to generate bacmid DNA, and transfected into Sf9 insect cells to produce recombinant baculoviruses (rBVs). The amplified rBVs are then used in High Five insect cells to express HA and M1 proteins, resulting in VLP formation (Kong et al., 2024). The use of such systems reduces the BSL (Lai et al., 2019).

VLPs are considered promising vaccine candidates due to their ability to elicit robust immune responses. The repetitive surface display of viral proteins in their native conformations triggers strong T and B cell-mediated immunity (Tariq et al., 2022). However, challenges in VLP production persist, particularly in achieving efficient assembly and optimizing transduction parameters (Gupta et al., 2023). In the specific context of studying H5N1 virus interactions with lung epithelial cells, particularly in relation to the glycocalyx, the complexity and time-intensive nature of VLP production make it less feasible.

### 6.5 DNA origami-based virus-like particles

An emerging and time-efficient alternative for creating synthetic viruses is DNA origami, a technique from DNA nanotechnology. This method allows for the bottom-up fabrication of highly defined nanostructures, ranging from tens of nanometers to sub-micrometers, thus providing a versatile tool for nanoscale engineering (Dey et al., 2021).

The DNA origami process uses a long single-stranded DNA (ssDNA) scaffold that is folded into intricate shapes with the aid of short oligonucleotide “staples.” These staples hybridize with complementary sequences on the scaffold, enabling the creation of complex nanostructures with high precision and quantitative yield. With the help of this technique polyhedral wireframe nanostructures were developed, offering precise control over two- and three-dimensional designs (Hong et al., 2017).

Functionalization of DNA origami typically involves post-assembly hybridizations, where single-stranded DNA overhangs on the nanostructure bind complementary nucleic acid strands linked to target conjugates. This approach allows orthogonal and programmable attachment of various biomolecules, including nucleic-acid-modified proteins, peptides, lipids, and dyes, with high site-specificity and efficiency (Knappe et al., 2023).

Recent advancements by Knappe et al. (2021) and Wei et al. (2024) have showcased the potential of DNA origami for creating synthetic DNA origami-based virus-like particles. They engineered an icosahedral DNA-origami capsid that could be functionalized post-assembly using click chemistry, enabling the integration of carbohydrates, small molecules, peptides, or proteins. By incorporating H and N proteins, this method offers promising applications in synthetic virology and vaccine development. A visual representation of the concept by Knappe et al. (2021) is seen in **Figure 4E**.

Although structural stability in DNA origami can be improved through strategies such as PEGylation (as demonstrated by Knappe and colleagues in 2021) (Knappe et al., 2021), degradation by nucleases over time remains a limitation. Addressing this issue is essential for broader adoption in studies such as H5N1 interactions with lung epithelial cells and the glycocalyx.

## 7 Visualization, tracking, and imaging techniques

Each virus model has distinct advantages and limitations. The choice of model depends on laboratory resources, visualization methods, target structures, and whether multiplexed staining is required. Depending on your chosen virus model, different challenges arise when visualizing the interplay and interactions between the virus, cells, and GAGs.

### 7.1 General challenges

The visualization of the interaction between viruses and GAGs in 3D cell culture systems presents a significant challenge on multiple levels.

The first challenge is to identify an optimal labeling strategy that allows studying the virus-GAG-interactions that are present on the cells. The chosen labeling strategy must minimize its impact on the infectivity of the virus and also ensure that it does not affect the natural function of the GAGs (Liu et al., 2023; Perez et al., 2023). As the objective is to track the virus, it is also necessary to consider the photostability of the fluorophores to allow the use of higher laser power and longer illumination time with the aim of maximizing the tracking time (Liu et al., 2020; Liu et al., 2023). Furthermore, higher photostability allows the selection of imaging techniques that require many photons for improved spatial resolution (Schmidt et al., 2021). With regard to cell labeling, a strategy that also works well for 3D cell culture must be selected.

The second challenge is to select the optimal tracking and imaging technique (Liu et al., 2020). There are many techniques that can be used for single virus tracking (SVT). The optimal technique should be capable of fulfilling several criteria in order to be considered for our intended use case (Liu et al., 2020). The selected technique must possess a high degree of spatiotemporal resolution, allowing for the simultaneous tracking of the virus and high-resolution imaging of the cells and GAGs (Liu et al., 2020; Johnson et al., 2022). In order to label these three structures simultaneously, it is also necessary to consider the use of multi-color imaging (Liu et al., 2020). Moreover, the selected technique must be capable of supporting live cell imaging (Liu et al., 2023).

An additional challenge that arises at each stage is the usability of traditional labeling and imaging/tracking techniques in 3D cell culture systems (Möckel et al., 2022). Verification of the efficacy of traditional labeling protocols poses a challenge as the labels must reach their intended targets which may be more complex in 3D cell culture. Therefore, it is essential to ensure that the penetration depth is sufficient (Möckel et al., 2022).

### 7.2 Labeling strategies

**Figure 5:**
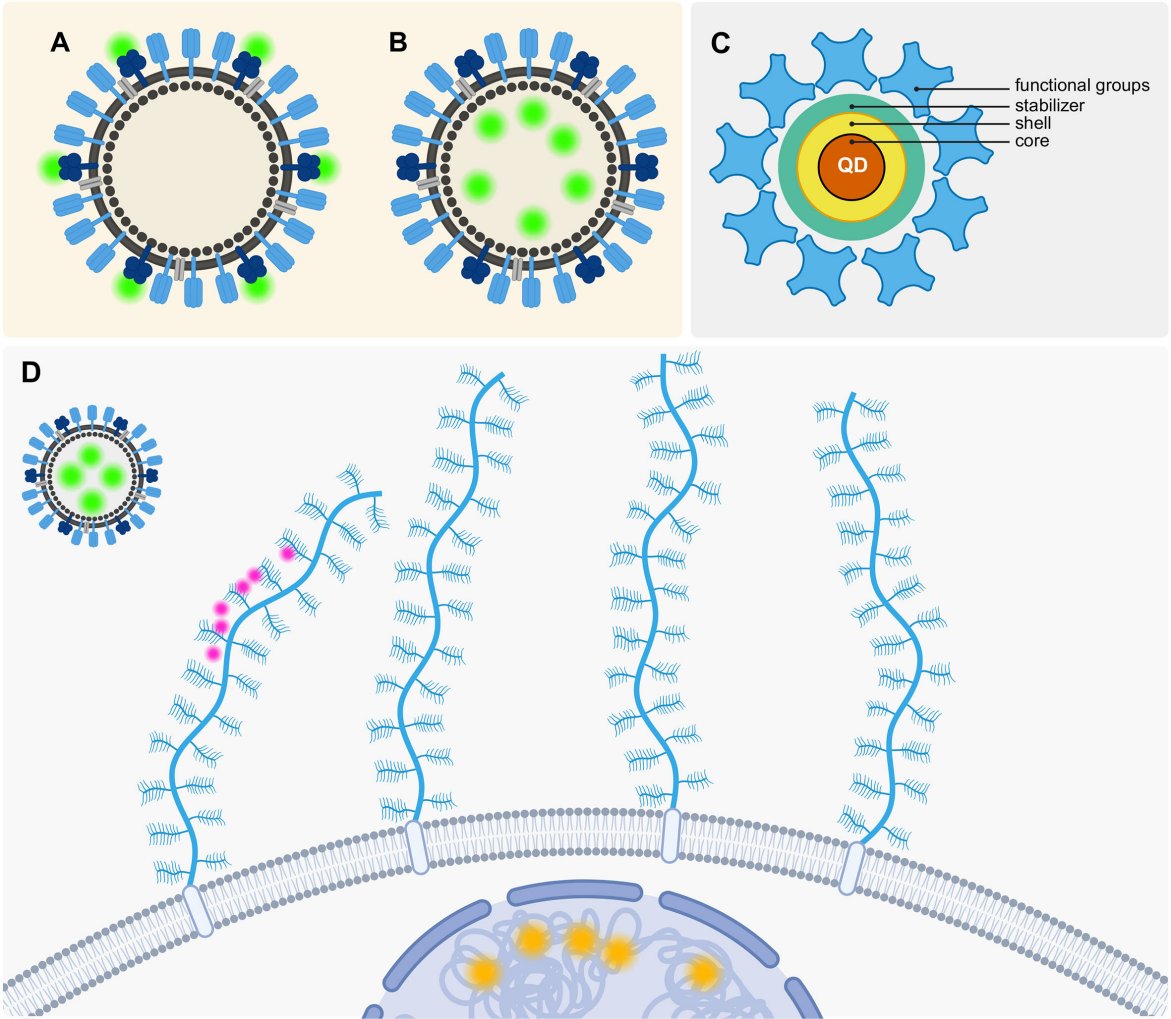
Labeling strategies to visualize the interaction between VLPs, GAGs and cells. **(A)** Outer labeling of a VLP at membrane proteins. **(B)** Inner labeling of the VLP. **(C)** Schematic QD consisting of its core, shell and stabilizer, and additionally added functional groups. **(D)** “Ideal” three color labeling strategy (Orange: DNA/RNA staining to visualize the cells, Green: QD labeling to visualize the VLPs, Pink: Labeling GAGs that are attached to proteoglycans (blue). Created with Biorender.com.

To study the interaction between GAGs and IAV in three-dimensional cell culture systems, it is essential to implement an effective labeling strategy. In an optimal scenario, the virus, GAGs, and cells would be labeled in a manner that does not impair their natural functions while providing a photostable signal for effective tracking and high-resolution multicolor imaging. The initial decision to be made is how to label your virus model. For example, a synthetic virus can be labeled internally or externally on the viral envelope as illustrated in **Figures 5A, B**. However, this does not apply to AuNPs since they do not have a membrane or inside.

One way to achieve a good visualization of the VLP is through a dual labeling strategy where an inner and outer labeling is employed simultaneously to be able to visualize the interaction of viruses and their host cells and track the viral infection (Liu et al., 2012). However, an outer labeling may impede the interactions between GAGs and the VLPs. Therefore, internal labeling would be a more optimal approach in this context. It is possible to track VLP solely through internal labeling, a technique that has already been demonstrated to be effective in both two-dimensional and three-dimensional cell culture systems (Johnson et al., 2022).

In addition to determining the optimal labeling strategy for the VLP, it is essential to identify the most suitable technique and fluorophore for this specific application. Three common methods for labeling include: fluorescent proteins, organic dyes, and quantum dots (QDs) (Wang et al., 2021). Each of these methods has distinct properties, which can be seen in **Table 3**. Fluorescent proteins encompass a variety of labeling strategies, including genetically encoded GFP. Organic dyes, such as Alexa Fluor dyes, can be utilized in conjunction with antibody staining.

**Table 3:** Comparison of fluorescent labels used for SVT.

	Quantum dots	Fluorescent protein	Organic dye
Probe size	1–20 nm	2 nm	1–2 nm
Target sites	Proteins, lipids, nucleic acids	Proteins	Proteins, lipids, nucleic acids
Photostability	>1 h	100 ms	1–20 s
Brightness	High	Low	Moderate
Usefulness for SVT	Good, but limited by blinking	Moderate photobleaching	Moderate photobleaching

Adapted from (Wang et al., 2021).

In contrast, QDs function in a distinct manner as illustrated in **Figure 5C**. A QD is a nanoscale semiconductor composed of multiple layers. The middle of the QD consists of an inorganic core nanocrystal which is often made up of cadmium selenide and is surrounded by a shell that is commonly made up of zinc sulfide (Wang et al., 2021). The organic coatings which include polymers, PEG, lipids, and small molecules, serve as stabilizers for the QDs (Wang et al., 2021). The outer layer of the QD is functionalized to allow for the flexible and specific labeling of different structures. The QDs’ diverse properties render them an attractive option for bioimaging applications. Their high brightness, large Stokes shift, broad excitation spectrum, size-tunable emission, and photostability make them particularly well-suited for single-virus tracking (Wang et al., 2021).

For the purposes of our case, it would be advisable to employ an inner virus labeling technique utilizing QDs. A protocol for the implementation of this technique has already been demonstrated to be effective (Liu et al., 2023). The inner labeling of the VLPs with QDs can be achieved with relative ease by utilizing electroporation (Liu et al., 2023), alternatively liposomes can also be loaded with QDs, for example via thin-film hydration or Reverse-phase evaporation (Beloglazova et al., 2013).

Two options for the labeling of cells are actin or DNA staining, which both can be used to visualize the cells for SVT (Johnson et al., 2022).

The labeling of GAGs is a more complex issue as the objective is to achieve labeling without compromising the natural function of these molecules. The majority of modifications, such as fluorescent labeling of GAGs, are randomly distributed along the polysaccharide backbone with no control over the binding position (Perez et al., 2023). Single-site functionalization of GAGs is a viable approach to labeling the polysaccharides without compromising their natural behavior. This can be achieved by targeting specific points within the polymeric structure (Perez et al., 2023). However, these functionalizations are currently limited to the (pseudo)-reduction of the ends of polysaccharide chains. Other site-specific modifications remain a goal for future research (Perez et al., 2023). Thus, labeling at the reducing end of GAGs would be the optimal approach for introducing a fluorescent label without compromising the functionality of GAGs. A study from 2019 managed to image the glycocalyx with 10–20 nm precision through metabolic incorporation of N-azidoacetylgalactosamine (GalNAz) to introduce azide groups for the probe conjugation and through the periodate-mediated oxidation of sialic acids for the introduction of aldehyde groups. While Sias label at the non-reducing end and thus would be suboptimal, N-acetylgalactosamine residues are found at the reducing end and would be more suitable for our goal (Möckl et al., 2019).

A recent paper proposed a different approach to label the ECM with a cell-impermeable small-molecule fluorophore called Rhobo6. It reversibly binds to glycans with a low affinity which also switches the fluorophore on and causes a redshift (Fiore et al., 2025).

**Figure 5D** depicts a schematic representation of our theoretical, optimal labeling strategy. It is our objective to label the cells, VLPs, and GAGs in three distinct colors. Furthermore, we propose to label the VLPs with QDs in order to ensure optimal photostability for SVT. The cells may be labeled using traditional actin- or intercalating DNA/RNA stains. As described previously, labeling GAGs without impacting their function will be more complex and will require the development of a custom strategy that could, for example, be based on bioorthogonal chemistry approaches like the aforementioned labeling of the non-reducing ends of GAGs through the periodate-mediated oxidation of sialic acids (Möckl et al., 2019).

### 7.3 Imaging and tracking

Once the optimal labeling strategy has been selected, the imaging and tracking technique that aligns with the labeling strategy and cell culture system must be chosen. The system must possess several essential properties to fulfill our requirements. These include the capacity for live cell imaging, a sufficient penetration depth for imaging 3D cell cultures, multicolor imaging, and the capability to image in 3D as our research employs a 3D cell culture system and necessitates a comprehensive understanding of the interactions within this system.

There are two principal approaches to track VLPs. One is an imaging-based approach where a time series is recorded and subsequently the particles within these images can be tracked (Liu et al., 2020). The second approach is a more direct technique where an active feedback loop is employed within the setup to track single particles (Liu et al., 2020). In contrast to the approach mentioned before, only a trajectory is being recorded rather than an image (Liu et al., 2020).

One of the imaging-based techniques that can readily achieve large excitation depths and image a substantial field of view is widefield microscopy. However, widefield microscopy is not capable of achieving a high resolution and background noise from out-of-focus illumination is a common problem. Furthermore, due to the excitation depth, three-dimensional imaging is not often performed (Lichtman and Conchello, 2005; Liu et al., 2020). These limitations rule out the use of widefield microscopy for our proposed use case.

Total internal reflection fluorescence (TIRF) microscopy can be employed to achieve a higher signal-to-noise ratio. TIRF employs an evanescent wave for excitation at the glass-sample interface at a critical angle where the light cannot penetrate deeply into the sample and is instead reflected entirely, thereby enhancing the signal-to-noise ratio (Johnson et al., 2012; Liu et al., 2020).

In order to achieve a higher resolution compared to the camera-based techniques, confocal microscopes can reduce the out-of-focus light and enhance the signal-to-noise ratio. They employ point illumination and a pinhole in front of the detector to eliminate out-of-focus light, thereby achieving higher resolution than that of widefield and TIRF microscopy (Naredi-Rainer et al., 2013; Liu et al., 2020). The two principal confocal techniques are laser scanning and spinning disk confocal microscopy. Spinning disk confocal microscopes offer a higher imaging speed rendering them more suitable for SVT than laser scanning confocal microscopes. Confocal microscopes can be easily modified with a piezo-z-scanning stage to facilitate three-dimensional tracking in live cells (Liu et al., 2020).

Light sheet microscopy represents an alternative approach since confocal microscopes require a significant amount of time to image a full frame and also cause photobleaching. This method uses a focused plane of light to illuminate the sample and the resulting emission is collected and imaged (Krzic et al., 2012). Light sheet microscopy permits faster imaging and deeper three-dimensional imaging at single-cell resolution, which enables SVT (Liu et al., 2020).

To further enhance the imaging resolution, super-resolution imaging techniques such as photo-activated localization microscopy (PALM) or stochastic optical reconstruction microscopy (STORM) can be employed (Betzig et al., 2006; Rust et al., 2006). As viruses are often smaller than the diffraction limit, super-resolution imaging would increase the localization precision of our target. However, this approach often introduces additional complications, including low temporal resolution, high optical toxicity, and a more complex post-processing procedure (Liu et al., 2020).

The most promising super-resolution technique may be a combination of single-particle tracking and PALM or STORM. In this approach, individual particles are tracked by activating only a small number of fluorophores, which are then tracked until they photobleach, initiating a new cycle (Manley et al., 2008; Liu et al., 2020). Thus, many trajectories can be collected, but they are often relatively short as these photoactivatable dyes often have poor photostability.

All of these methods share a common limitation: they either possess a high temporal resolution or a high spatial resolution. But with these methods, multiple particles can be tracked at once, in contrast to setups in which the particles are directly tracked and only one particle can be tracked at a time (Liu et al., 2020).

These “direct” single particle tracking (SPT) techniques employ a feedback loop to adjust the localization position. Examples of such methods include orbital tracking and MINFLUX, a nanoscopy technique that achieves single-digit nanometer localization precision and microsecond range tracking (Wells et al., 2010; Liu et al., 2020; Schmidt et al., 2021). While MINFLUX tracking is a promising method for the future, it is currently not possible to simultaneously image the surroundings of the tracked VLP. Consequently, it is not a viable option for our SVT case.

Alternatively, both approaches can be combined by utilizing an active feedback loop to track the VLP while simultaneously employing a two-photon laser setup for three-dimensional scanning. This approach allows for high-spatial-resolution imaging while simultaneously maintaining high temporal resolution (Johnson et al., 2022). This setup, called 3D Tracking and Imaging Microscopy (3D-TrIm) consists of two separate parts: 3D single-molecule active real-time tracking (3D-SMART) and 3D Fast Acquisition by z-Translating Raster (3D-FASTR) (Johnson et al., 2022). In the same way as other super-resolution techniques, 3D-SMART does not record an image but the position of the localized molecule, yielding recorded trajectories with a high temporal resolution (Johnson et al., 2022). To also add context to the tracked molecule, 3D-FASTR allows volumetric imaging of the surrounding area (Johnson et al., 2022). The researchers achieved a localization precision of 20 nm in the xy direction and 80 nm in the z direction and a temporal resolution of 1000 localizations per second which enabled them to track VLPs with high spatial and temporal resolution while simultaneously tracking their surroundings in 3D (Johnson et al., 2022). Most other techniques described above either lack the spatial or the temporal resolution or are not capable of imaging the surroundings of the tracked particle, which is necessary to study the interactions of the tracked virus with the labeled GAGs and cells. This is why their approach represents a synthesis of the strengths of imaging-based and direct tracking-based methods while also enabling SVT in 3D cell cultures (Johnson et al., 2022).

Furthermore, the two-photon laser scanning setup enables superior three-dimensional imaging with reduced background noise in comparison to traditional laser-scanning setups. This is achieved by minimizing photobleaching and constricting the excitation to the focal plane. Two-photon lasers also permit deeper penetration into the samples, rendering them optimal for three-dimensional imaging (Denk et al., 1990).

This configuration is arguably the optimal choice for our case; however, it necessitates a highly customized setup which is not feasible for all laboratories. For laboratories that lack the resources to construct a setup, commercial options like the ONI nanoimager may be more suitable. These systems are capable of super-resolution imaging and SPT.

## 8 Discussion

IAVs remain a major threat to human health. Viral evolution continues, as do efforts to understand the molecular events that occur during viral infection. Understanding how a virus enters its host cell is crucial for prevention and research. The first H5N1-related casualty has just been reported in the USA, although the risk of human-to-human transmission remains low for now (Parkinson and Halsey, 2025). This comprehensive review explores crucial factors influencing IAV entry into the respiratory system.

Glycans, particularly GAGs, play a crucial role in viral entry. As components of the glycocalyx, they can serve as initial attachment factors, mediating low-affinity interactions that facilitate subsequent, more specific viral binding.

To investigate these interactions, various 3D lung cell culture models are available, each with distinct advantages and limitations depending on the research question and available resources. Among these models, the ALI-OTE model stands out as a promising approach, integrating structural and biochemical components to better simulate *in vivo* conditions for studying IAV infection and the role of GAGs.

Additionally, different synthetic virus models were discussed that serve as a safe alternative to study IAV-glycan-cell interactions. These models lack the viral genome while preserving key structural and epitope features. Various nanoarchitectures like AuNPs, SAPs, liposomes, VLPs, and DNA origami show potential to mimic viruses.

Accurately visualizing virus-GAG interactions in 3D cell culture systems requires careful selection of labeling strategies and imaging techniques to preserve the natural functions of the studied components while achieving high spatiotemporal resolution. Labeling VLPs with quantum dots and labeling the non-reducing ends of GAGs seems like the best approach so far. From the imaging perspective, 3D-TrIm microscopy looks like the most promising solution for tracking and imaging the virus-GAG interactions. However, further developments and custom setups may be necessary to address remaining challenges and improve accessibility for broader applications.
